# Solitary Brain Mass in a Patient with Seizures: An Unexpected Infectious Etiology

**DOI:** 10.3390/diseases6030054

**Published:** 2018-06-22

**Authors:** Mohammed Raja, Jose Armando Gonzales Zamora, Ali Hassoun

**Affiliations:** 1 Division of Infectious Diseases, Department of Medicine, University of Miami, Miller School of Medicine, Miami, FL 33136, USA; moraja87@gmail.com; 2 Division of Infectious Diseases, Department of Medicine, Huntsville Hospital, Huntsville, AL 35801, USA; ali_hasoun@yahoo.com

**Keywords:** neurocysticercosis, seizures, brain mass, *Taenia solium*

## Abstract

Neurocysticercosis is a parasitosis caused by the larval stage of the pork tapeworm *Taenia solium.* The diagnosis is challenging as morphology on neuroimaging can be inconclusive and serology is frequently negative. We describe the case of a 24-year old Hispanic man who presented with seizures and loss of consciousness. Magnetic resonance imaging (MRI) showed a cystic mass in right frontal lobe. Work-up that included body computed tomography (CT) scan and Western blot serology for *Echinococcus* and cysticercosis was unrevealing. He underwent craniotomy with resection of the mass. Histopathology showed fragments of *Taenia solium*. He was treated with albendazole for 14 days. No further seizures were noted at 6-month follow-up.

**Figure 1:**
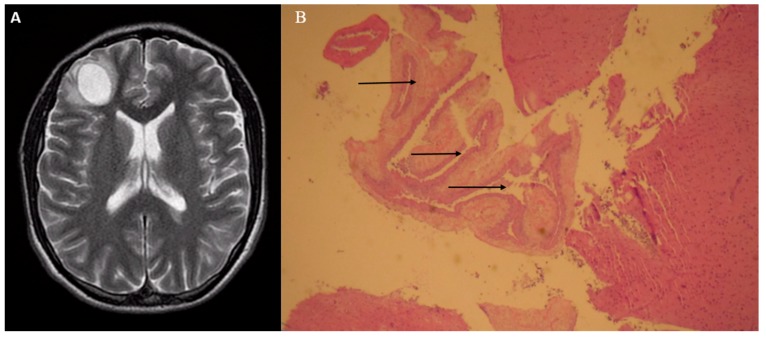
A 24-year old Hispanic man was found unresponsive at home after having multiple episodes of complex partial seizures. He was originally from Mexico and traveled there three weeks prior to hospital admission. His neurological exam did not reveal any focal deficits. He was intubated for airway protection and treated with fosphenytoin. A magnetic resonance imaging (MRI) with gadolinium contrast was ordered. T2-weighted imaging showed a cystic lesion of 2.2 × 2.5 cm in diameter with surrounding edema in right frontal lobe (**A**). Western blot serology for *Echinococcus* and cysticercosis was negative. Due to the concern for a neoplastic process and negative serology for parasitosis, computed tomography (CT) of chest, abdomen, and pelvis was ordered, which was unremarkable. The patient underwent craniotomy with resection of the mass. On gross inspection, the mass appeared cystic. Histopathology with hematoxylin and eosin (H&E) staining revealed benign brain parenchyma with degenerated fragments of a parasite consistent with *Taenia solium* (**B**). The patient was treated with albendazole for 14 days. No further seizures were noted at 6-month follow-up. Neurocysticercosis is a parasitosis caused by the larval stage of the pork tapeworm *Taenia solium*. It can be associated with various neuroimaging findings including solitary mass, which can be a diagnostic dilemma. Neurocysticercosis typically involve the cerebral hemispheres, with lesions found at the gray-white matter junction. In individuals with multiple lesions, the basal ganglia, cerebellum and brainstem may be affected. The appearance of these brain lesions on neuroimaging depends on their stage of involution. Four stages can be recognized on CT or MRI: vesicular, colloidal, granular nodular, and calcified lesions [1]. Live vesicular cysts are small and rounded lesions with little or no pericystic edema, not enhanced with contrast. The cyst fluid usually demonstrates the same signal intensity of cerebrospinal fluid on all MRI sequences. In this stage, the scolex is frequently identified as an internal asymmetric nodule in the cyst. Fluid-attenuated inversion recovery (FLAIR) imaging can improve the visualization of the T2-weighted hyperintense scolex [1]. After the degenerative process becomes established (colloidal stage), the cysts show poorly defined borders with surrounding edema. MRI usually reveals ring contrast enhancement [2]. At this stage, absence of diffusion restriction is evident on diffusion-weighted (DW) imaging, which helps to distinguish these lesions from abscesses. In the granular nodular stage, the lesions are smaller and imaging demonstrate hyperintense rims, representative of gliosis [2]. Calcified nodules are the end stage of cysticercal degeneration and can be seen as hyperdense lesions with no associated edema or enhancement. CT is the most sensitive imaging to identify lesions at this stage [1]. In terms of clinical manifestations, patients may remain asymptomatic, particularly with viable non-degenerating cysts; however, seizures are the most common presentation in cases of degenerating cysts that produce an injurious inflammatory response [3]. In patients with single parenchymal lesions, the diagnosis is challenging, as morphology on neuroimaging can be inconclusive and serology is commonly negative [4]. In endemic regions, neurocysticercosis may be confounded with other infections that affect the brain parenchyma such as tuberculosis, owing to similar radiographic appearance [5]. Albendazole is the treatment of choice, which should be administered with corticosteroids to reduce the inflammation associated with dying organisms [6,7]. Our case highlights the importance of considering neurocysticercosis in the differential diagnosis of solitary brain masses, especially in patients coming from endemic countries. The radiographic features of this condition vary depending on the stage of involution of the cysts. They are best visualized by MRI in early stages, while CT is the preferred imaging at the end stage of cysticercal degeneration.
